# The PRINCIPLE randomised controlled open label platform trial of hydroxychloroquine for treating COVID19 in community based patients at high risk

**DOI:** 10.1038/s41598-025-09275-6

**Published:** 2025-07-04

**Authors:** F. D. Richard Hobbs, Jienchi Dorward, Gail Hayward, Ly-Mee Yu, Benjamin R. Saville, Christopher C. Butler, the PRINCIPLE Investigators, F. D. Richard Hobbs, Jienchi Dorward, Gail Hayward, Ly-Mee Yu, Benjamin R. Saville, Christopher C. Butler, Jared Robinson, Charlotte Latimer Bell, Oghenekome Gbinigie, Oliver Van Hecke, Emma Ogburn, Nicholas P. B. Thomas, Philip H. Evans, Monique Andersson, Emily Bongard, Aleksandra Borek, Jenna Grabey, Victoria Harris, Mona Koshkouei, Mahendra G. Patel, Nick Berry, Michelle A. Detry, Christina T. Saunders, Mark Fitzgerald

**Affiliations:** 1 Nuffield Department of Primary Care Health Sciences, University of Oxford, Oxford, UK; 2 Berry Consultants, Austin, TX USA; 3 Department of Biostatistics, Vanderbilt University School of Medicine, Nashville, TN USA; 4 Windrush Medical Practice, Whitney, USA; 5 Clinical Research Network, University of Exeter and National Institute for Health and Care Research, APEx, UK; 6 Brighton and Sussex Medical School, University of Sussex, Brighton, UK; 7 School of Pharmacy and Medical Sciences, University of Bradford, Bradford, UK

**Keywords:** Primary health care, SARS-COV-2, Serious COVID, Repurposed medicines, Hydroxychloroquine, Diseases, Infectious diseases, Viral infection

## Abstract

Early on in the COVID-19 pandemic, we aimed to assess the effectiveness of hydroxychloroquine on reducing the need for hospital admission in patients in the community at higher risk of complications from COVID-19 syndromic illness (testing was largely unavailable at the time, hence not microbiologically confirmed SARS-CoV-2 infection), as part of the national open-label, multi-arm, prospective, adaptive platform, randomised clinical trial in community care in the United Kingdom (UK). People aged 65 and over, or aged 50 and over with comorbidities, and who had been unwell for up to 14 days with suspected COVID-19 were randomised to usual care with the addition of hydroxychloroquine, 200 mg twice a day for seven days, or usual care without hydroxychloroquine (control). Participants were recruited based on symptoms and approximately 5% had confirmed SARS-COV2 infection. The primary outcome while hydroxychloroquine was in the trial was hospital admission or death related to suspected COVID-19 infection within 28 days from randomisation. First recruitment was on April 2, 2020, and the hydroxychloroquine arm was suspended by the UK Medicines Regulator on May 22, 2020. 207 were randomised to hydroxychloroquine and 206 to usual care, and 190 and 194 contributed to the primary analysis results presented, respectively. There was no swab result available within 28 days of randomisation for 39% in both groups: 107 (54%) in the hydroxychloroquine group and 111 (55%) in the usual care group tested negative for SARS-Cov-2, and 13 (7%) and 11 (5%) tested positive. 13 participants, (seven (3·7%) in the usual care plus hydroxychloroquine and six (3.1%) in the usual care group were hospitalized (odds ratio 1·04 [95% BCI 0·36 to 3.00], probability of superiority 0·47). There was one serious adverse event, in the usual care group. More people receiving hydroxychloroquine reported nausea. We found no evidence from this treatment arm of the PRINCIPLE trial, stopped early and therefore under-powered for reasons external to the trial, that hydroxychloroquine reduced hospital admission or death in people with suspected, but mostly unconfirmed COVID-19.

**Supplementary Information:**

The online version contains supplementary material available at 10.1038/s41598-025-09275-6.

## Introduction

Hydroxychloroquine for treating COVID-19 was widely promoted early on in the pandemic and over 100 trials evaluating hydroxychloroquine were quickly registered. This was supported by in vitro studies that found hydroxychloroquine has anti SARS-CoV-2 activity, although some data was later questioned^1,2^. Various mechanisms of action have been suggested, including a hydroxychloroquine induced reduction in the glycosylation of host cell surface enzymes preventing viral attachment^3^, and inhibition of the production of certain pro-inflammatory cytokines that are mediators of acute respiratory distress syndrome^4^. Hydroxychloroquine is generally safe and well tolerated^5,6^, cheap and widely available. If effective in speeding recovery and preventing hospital admissions, re-positioning hydroxychloroquine for community treatment of COVID-like-illness could be rapidly scaled up with widespread impact.

Observational studies early in the pandemic found associations between hydroxychloroquine use and reduced mortality^7,8^. However, other large-scale studies found no meaningful benefit from hydroxychloroquine in those admitted to hospital, but that the drug did not result in worse cardiovascular or other outcomes^9–11^. Some therapies might have greater effectiveness if initiated early in the illness course: two trials, one placebo controlled^12^ and one open^13^ in non-hospitalised patients found non-statistically significant benefit in terms of measures of symptom duration and severity from early treatment of COVID-19 with hydroxychloroquine. Neither trial found a statistically significant difference in the small number of hospitalisations between groups^12,13^. A Cochrane review that included 10 trials in hospitalised patients and two in outpatients of hydroxychloroquine/ chloroquine treatment of COVID-19 found no benefits on mortality and viral clearance, but only included trials up to September 2020^14^. The control condition was usual care in ten trials and placebo in one. While early reviews of trials found evidence of benefit, when trials with questionable rigour were removed, revised metanalyses including higher quality trial evidence, generally found no evidence of benefit. A systematic review found no effect on mortality or hospitalization in participants with COVID-19^15^ and an individual participant meta-analysis similarly showed no effect^16^.

We report findings of the effectiveness of hydroxychloroquine from the Platform Randomised trial of INterventions against COVID-19 In older peoPLE (PRINCIPLE), the United Kingdom (UK) National Urgent Public Health Priority platform trial of interventions for COVID-19 syndromic illness in people in the community at higher risk of an adverse illness course. This evaluation took place before testing and vaccination became widely available, hence the focus on the syndrome.

## Methods

This is an open-label, multi-arm, prospective, adaptive platform, randomized clinical trial in community care. A “platform trial” is an adaptive clinical trial in which multiple treatments for the same disease are tested simultaneously. Participants are randomised to either usual care or an intervention plus usual care. A master protocol defines prospective decision criteria to allow for dropping a treatment for futility, declaring a treatment superior, or adding a new treatment to be evaluated^17^. Here, we report outcomes from hydroxychloroquine, the first intervention included in the trial. The hydroxychloroquine arm was stopped early by the UK regulator because of safety concerns.

Ethical approval was obtained from the South Central-Berkshire Research Ethics Committee under authority of the United Kingdom Ethics Committee Authority under the Medicines for Human Use (Clinical Trials) Regulations 2004 on 22/03/2020 registration number (ISRCTN86534580). Online informed consent was obtained from all participants. The trial was conducted in accordance with the principles of the Declaration of Helsinki and the International Conference on Harmonization–Good Clinical Practice guidelines. The protocol is available online.

People in the community were eligible if they were aged 65 and over, or aged 50 and over with comorbidity, and with ongoing symptoms from suspected (in accordance with the UK’s National Health Service’s (NHS) syndromic case definition at the time) or PCR confirmed SARS-CoV-2 infection, and illness duration of up to a maximum of 14 days. At the time the trial first opened, testing for SARS-COV-2 infection was not widely available and so we considered syndromic illness. Co-morbidities required for eligibility in those aged 50 to 65 years were: known weakened immune system due to a serious illness or medication (e.g. chemotherapy); known heart disease and/or a diagnosis of high blood pressure; known asthma or lung disease; known diabetes; known mild hepatic impairment; and known stroke or neurological problem.

People were ineligible if they had a contraindication to hydroxychloroquine.

Participants were recruited, screened, and enrolled through participating general practices (over 800 practices were ‘green lit’ to recruit into the trial by May 22, 2020).

Participants were randomised to receive oral hydroxychloroquine 200 mg twice daily for seven days, or usual care without hydroxychloroquine. Usual Care in the NHS for suspected COVID-19 in the community at the time was largely supportive, with no recommendation for routine use of antibiotics unless bacterial pneumonia was suspected.

The first participant was recruited on the April 2, 2020. The hydroxychloroquine arm of the study was suspended on May 23, 2020, on the instruction of the Medicine and Healthcare Products Regulatory Authority (MHRA) and was not restarted. This MHRA policy was applied to all clinical trials of Hydroxychloroquine in the UK. Participants recruited up to that point were followed up according to the protocol.

Participants responded to questions about their symptoms, medications use and help seeking behaviour in a daily online diary for 28 days after randomisation. This was supplemented with telephone calls on days 7, 14 and 28, where a minimum data set was collected if the online diary questions had not been answered. Participants were encouraged to nominate a trial partner to help provide follow up data and be contacted if the participant was unable to complete the daily diary for themselves.

We aimed to provide all participants with the opportunity to take a self-swab to send to a central laboratory for PCR testing for SARS-CoV-2 infection but due to nationwide supply issues in the early weeks of the pandemic, swabs were unavailable for this purpose for most of the trial period of this intervention. Hence, people were included based on an acute syndromic COVID-like illness, largely unconfirmed by testing.

The primary outcome was hospitalisation or death within 28 days of presumed COVID infection at the time that hydroxychloroquine was in the trial.

Secondary outcomes include time to first reported recovery, time to sustained recovery (date participant first reports feeling recovered and subsequently remains well until 28 days), time to initial alleviation of symptoms, time to sustained alleviation of symptoms (date participant first reports all symptoms as minor or none, with no subsequent increase in symptoms until 28 days), time to initial reduction of severity of symptoms, hospital assessment without admission; oxygen administration; Intensive Care Unit admission; mechanical ventilation (components of the WHO Ordinal Scale); duration of hospital admission; contacts with health services; consumption of antibiotics; effects in those with a positive test for COVID-19 infection; and the WHO Well-being Index. We included secondary outcomes that captured sustained recovery due to the recurrent nature of COVID-19 symptoms.

Approximately 1,500 participants per arm (3,000 participants total if only a single intervention vs. Usual Care) were required to provide 90% power for detecting a 50% reduction in the relative risk of hospitalisation and/or death. Assuming a median time to recovery of nine days in the Usual Care arm, approximately 400 participants per arm (800 participants total if only a single intervention vs. Usual Care) were required to provide 90% power for detecting a difference of 2 days in median recovery time. However, due to the suspension of accrual to the hydroxychloroquine arm, the sample size for the present analysis was limited to 207 participants allocated to hydroxychloroquine, and 206 to usual care. The concurrent analysis results are based on 190 randomized to hydroxychloroquine and 194 randomized to usual care.

After an online consent process and eligibility check using primary care medical records, participants were randomized using a secure, in-house web-based randomization system called Sortition. Initially, randomization was fixed at 1:1 for a comparison between hydroxychloroquine and usual care, with stratification by age (< 65/≥ 65), and comorbidity (yes/no). There were no additional interventions other than hydroxychloroquine available prior to 22 May 2020, hence randomization probabilities remained 1:1 for hydroxychloroquine versus Usual Care.

All outcome analyses were conducted using the concurrent analysis population, i.e. hydroxychloroquine participants versus concurrently randomized controls. The primary outcome was analysed using a Bayesian logistic regression model. Time to first reported recovery was analysed using a Bayesian piecewise exponential model. Other time-to-event outcomes were analysed using Cox proportional hazard models, and binary outcomes were analysed using logistic regression. We also conducted sensitivity analyses for the primary outcomes using all participants randomized to 30th November 2020, with the time drift effect accounted for via the prior distributions. Safety analyses were conducted on the treatment that the participants received.

UK Research and Innovation and the Department of Health and Social Care through the National Institute for Health Research as part of the UK Government’s Urgent Public Health Priority research funding. The International Standard Randomised Controlled Trial Number is ISRCTN86534580.

## Results

First recruitment was on April 2, 2020, but the hydroxychloroquine arm was suspended by the UK Medicines Regulator on May 22, 2020. 207 were randomised to hydroxychloroquine and 206 to usual care. 7 participants randomised to hydroxychloroquine and 2 randomised to usual care were excluded due to ineligibility. A further 4 hydroxychloroquine participants and 3 usual care withdrew consent for notes review and did not provide diary data. 6 hydroxychloroquine and 5 usual care did not withdraw consent but completed no diaries and 2 usual care reported a time to recovery of 0 days. This left 194 and 190 evaluable respectively. The average age was 60 years, and median duration of illness prior to randomization was 5 days. There was no swab result available for 39% in both groups, 107 (54%) in the hydroxychloroquine group and 111 (55%) in the usual care group tested negative, and 13 (7%) and 11 (5%) tested positive (Table 1).

**Table 1:** Baseline characteristics of all randomized participants excluding those found to be ineligible by randomised arm.

	HCQ (*N* = 197)	Usual Care (*N* = 202)	OVERALL (*N* = 399)
**Age**,** mean(SD)**	60.7 (7.8)	59.9 (7.6)	60.3 (7.7)
*≥ 65 years*	140 (71%)	144 (71%)	284 (71%)
*< 65 years*	57 (29%)	58 (29%)	115 (29%)
**Female**,** n(%)**	109 (55%)	112 (55%)	221 (55%)
**Ethnicity**,** n(%)**^**a**^			
*White*	121 (61%)	139 (69%)	260 (65%)
*Mixed background*	14 (7%)	9 (4%)	23 (6%)
*South Asian*	3 (2%)	3 (1%)	6 (2%)
*Black*	0 (0%)	2 (1%)	2 (1%)
*Other*	3 (2%)	4 (2%)	7 (2%)
*Not recorded/Missing*,* n(%)*	56 (28%)	45 (22%)	101 (25%)
**Duration of illness prior to randomisation (days)**,** median (IQR)**	5 (3 to 10)	5 (4 to 8)	5 (3 to 9)
**Smoking status**,** n(%)**			
*Current smoker*	30 (15%)	37 (18%)	67 (17%)
*Former smoker*	69 (35%)	63 (31%)	132 (33%)
*Never smoker*	92 (47%)	97 (48%)	189 (47%)
*Missing*	6 (3%)	5 (2%)	11 (3%)
**Swab result within 28 days of randomisation**,** n(%)**			
*Negative*	107 (54%)	111 (55%)	218 (55%)
*Positive*	13 (7%)	11 (5%)	24 (6%)
*No swabresults available*	1 (1%)	1 (0%)	2 (1%)
*No swab test*	76 (39%)	79 (39%)	155 (39%)
**Comorbidity**,** n(%)**			
*No*	23 (12%)	22 (11%)	45 (11%)
*Yes*	174 (88%)	180 (89%)	354 (89%)
**Asthma**,** COPD**^**b**^ **or lung disease**,** n(%)**	89 (45%)	96 (48%)	185 (46%)
**Diabetes**,** n(%)**	25 (13%)	33 (16%)	58 (15%)
**Heart problems**,** n(%)**^**c**^	27 (14%)	37 (18%)	64 (16%)
**High blood pressure for which you are taking medications**,** n(%)**	98 (50%)	100 (50%)	198 (50%)
**Liver disease**,** n(%)**	4 (2%)	7 (3%)	11 (3%)
**Stroke or other neurological problem**,** n(%)**	22 (11%)	19 (9%)	41 (10%)
**Taking ACE inhibitor**,** n(%)**^**d**^	45 (23%)	54 (27%)	99 (25%)
*Missing*	2 (1%)	1 (0%)	3 (1%)
**Taken antibiotics since their illness started**,** n(%)**	23 (12%)	16 (8%)	39 (10%)
**Type of healthcare services contacted since their illness started**:			
**GP**^**e**^, **n(%)**	89 (45%)	76 (38%)	165 (41%)
**Other primary care services**,** n(%)**	14 (7%)	18 (9%)	32 (8%)
**NHS 111**,** n(%)**	65 (33%)	58 (29%)	123 (31%)
**A&E**^**f**^, **n(%)**	6 (3%)	3 (1%)	9 (2%)
**Other healthcare services**,** n(%)**	14 (7%)	16 (8%)	30 (8%)
**Baseline symptoms**			
**Fever**,** n(%)**			
*No problem*	85 (43%)	84 (42%)	169 (42%)
*Minor problem*	65 (33%)	73 (36%)	138 (35%)
*Moderate problem*	40 (20%)	42 (21%)	82 (21%)
*Major problem*	7 (4%)	3 (1%)	10 (3%)
**Cough**,** n(%)**			
*No problem*	28 (14%)	30 (15%)	58 (15%)
*Minor problem*	75 (38%)	74 (37%)	149 (37%)
*Moderate problem*	84 (43%)	81 (40%)	165 (41%)
*Major problem*	10 (5%)	17 (8%)	27 (7%)
**Shortness of breath**,** n(%)**			
*No problem*	52 (26%)	38 (19%)	90 (23%)
*Minor problem*	89 (45%)	101 (50%)	190 (48%)
*Moderate problem*	53 (27%)	57 (28%)	110 (28%)
*Major problem*	3 (2%)	6 (3%)	9 (2%)
**Muscle ache**,** n(%)**			
*No problem*	56 (28%)	65 (32%)	121 (30%)
*Minor problem*	78 (40%)	70 (35%)	148 (37%)
*Moderate problem*	45 (23%)	49 (24%)	94 (24%)
*Major problem*	18 (9%)	18 (9%)	36 (9%)
**Nausea**,** n(%)**			
*No problem*	142 (72%)	156 (77%)	298 (75%)
*Minor problem*	43 (22%)	39 (19%)	82 (21%)
*Moderate problem*	8 (4%)	4 (2%)	12 (3%)
*Major problem*	4 (2%)	3 (1%)	7 (2%)
**Well-being (WHO5 Questionnaire)**,** mean(SD)**^**g**^	46.0 (24.2)	47.5 (23.2)	46.8 (23.7)
*Missing*	21 (11%)	24 (12%)	45 (11%)

^a^Data on ethnicity were collected retrospectively via notes review.

^b^ COPD: Chronic obstructive pulmonary disease.

^c^E.g. angina, heart attack, heart failure, atrial fibrillation, valve problems.

^d^ACE inhibitors: Angiotensin-converting enzyme inhibitors, such as Ramipril, Lisinopril, Perindopril, Captopril or Enalapril.

^e^General practitioner (family medicine doctor).

^f^Accident and emergency.

^g^ Well-being is measured using the WHO well-being index which includes 5 items relating to well-being measured on a five point scale. A total score is computed by summing the scores to the five individual questions to give a raw score ranging from 0 to 25 which is then multiplied by 4 to give the final score from 0 representing the worst imaginable well-being to 100 representing the best imaginable well-being.

Of the 413 randomised to UC and HCQ, 9 were excluded due to ineligibility, 5 withdrew consent to follow-up and provided no outcome data, 2 recovered at day 0 and 13 were excluded due to lack of recovery information, leaving 384 overall (194 UC and 190 HCQ) providing primary outcome data (Table 1).

## Primary outcome

6 participants in the usual care group and 7 in the usual care plus hydroxychloroquine group were hospitalized (odds ratio 1.04 95% [95% BCI 0·36 − 3.00], probability (Pr) of superiority 0·47) (Table 2). The estimated benefit in hospitalisation rate was − 0.1% [95% BCI − 3.9–3.5%].

**Table 2:** Primary and main secondary outcomes.

Analysis between hydroxychloroquine and concurrent controls		
	Hydroxychloroquine	Usual Care
Hospitalisation/Death*		
Number(%)	7/190 (3·7%)	6/194 (3·1%)
Odds Ratio (95% credible interval)^a^	1.04 (0.36 to 3.00)	
Estimated difference in hospital rate	-0.001 (-0.038 to 0.035	
Probability of superiority of HCQ to Usual Care, %	46.7%	
Probability of meaningful effect^b^, %	11.2%	
**Time to Recovery**		
Number analysed	190	194
Median survival (IQR), days	7 (4 to 13)	9 (3 to 21)
Adjusted Hazard Ratio^5^ (95% confidence interval)	1·27 (1·03 to 1·55)	
Probability of superiority of HCQ to Usual Care, %	98.8%	
Probability of meaningful effect^c^, %	74.5%	

^*^Primary outcome.

^a^ Bayesian piecewise exponential regression adjusted for age and comorbidity.

^b^ Pre-defined clinically meaningful effect is defined as 2% points absolute reduction in HCQ compared to Usual Care.

^c^ Pre-defined clinically meaningful effect is defined as 1.5 days reduction in HCQ compared to Usual Care.

## Secondary outcomes

Time to first reported recovery was shorter in those allocated to hydroxychloroquine than usual care, with an observed reduction of two days and modelled median time to recovery benefit of 2.12 days (hazard ratio 1·27 [95% confidence interval (CI) 1.03-1·55], Pr (superiority) = 0·988 (Table 2). Probability of meaningful effect on this outcome was based on a difference of 1.5 days at the time when hydroxychloroquine was open.

There was no evidence of any difference in the WHO wellbeing score between the two arms at days 14 and 28, or any of the hospitalisation secondary outcomes (Table 3).

**Table 3:** Secondary outcomes.

	HCQ	Usual Care	Treatment effect (95% CI)	*P*-value
**Sustained recovery**,** n/N(%)**	135/190 (71.1%)	129/194 (66.5%)		
**Time to Sustained recovery**	18 (9 to not reached)	20 (10 to not reached)	1.21 [0.953 to 1.546] ^a^	0.12
Self-reported contact with ≥ 1 healthcare service	118/186 (63.4%)	124/186 (66.7%)	0.87 (0.57 to 1.34)^b^	0.53
GP reported contact with ≥ 1 healthcare service	133/175 (76.0%)	137/187 (73.3%)	1.17 (0.73 to 1.90)^b^	0.51
Prescription of antibiotics	17/171 (9.9%)	22/179 (12.3%)	0.79 (0.38 to 1.62)^b^	0.50
Hospital assessment without admission	12/190 (6.3%)	6/194 (3.1%)	2.11 (0.71 to 7.00)^c^	0.15
Oxygen administered	3/189 (1.6%)	1/194 (0.5%)	3.11 (0.25 to 164.23)^c^	0.37
Mechanical ventilation	0/189 (0.0%)	0/194 (0.0%)		
Intensive care unit admission	0/189 (0.0%)	0/194 (0.0%)		
Well-being (WHO5 Questionnaire), mean (SD)[n]				
At day 14	45.3 (23.4) [170]	42.6 (21.4) [164]	3.18 (-0.96 to 7.31)^d^	0.13
At day 28	53.2 (24.1) [166]	50.6 (22.7) [164]	3.05 (-1.11 to 7.22)^d^	0.15

^a^Cox proportional hazards regression adjusted for age, comorbidity at baseline, and duration of illness.

^b^ Logistic regression adjusted for age, comorbidity at baseline, and duration of illness.

^c^Unadjusted odds ratio.

^d^Mixed effect model adjusting WHO5 score, age, comorbidity at baseline, and time. Participant was fitted as a random effect.

There was no impact of the duration of illness prior to randomisation or the baseline illness severity score on the time to recovery. Estimates of treatment benefit were similar for those aged less than 65 years and those aged 65 years and older, as well as between those with and without comorbidity. Hydroxychloroquine benefited those with no swab result available (Adjusted HR 1.519 [1.039 to 2.219] but not the 24 participants with a positive swab result within five days of randomisation (Adjusted HR 1.169 [0.469 to 2.918] (Supplementary Fig. 3). There was only one serious adverse event (non-COVID related hospitalisation due to E-Coli bacteraemia and UTI), occurring in the usual care group. Nausea and vomiting is a common side effect of hydroxychloroquine, and there was a trend towards greater duration of nausea-vomiting in those randomized to hydroxychloroquine (Supplementary Fig. 1).

## Discussion

In this open-label, multi-arm, prospective, adaptive platform, randomised clinical trial of interventions for people with COVID-19 syndromic illness in the community at higher risk of complications, we found no evidence of reduced hospital admissions or deaths from hydroxychloroquine treatment. However, the hydroxychloroquine arm was closed early by the regulators due to factors external to the trial, and the number of these primary events was small and the study underpowered.

Hydroxychloroquine added to usual care reduced the secondary outcome of median time to first feeling recovered by two days in both the observed data and the model estimates. The lack of freely available testing and the shorter duration of illness suggests that participants recruited early in the trial may have been less severely ill or may not have been infected with SARS-COV-2. Indeed, of the 50.4% of participants who were tested, only 10.4% of these had a positive PCR test for SARS-CoV-2 infection. Hydroxychloroquine is known to have anti-inflammatory properties, so the reduction in time to recovery may be due to a modest general effect on respiratory illness, possibly due to its anti-inflammatory effect. Our pre-specified subgroup analysis which showed a greater effect on time to recovery in those with no swab result versus those testing positive for SARS-Cov-2 infections, suggesting the effect was not specific to COVID-19. There was a trend towards greater duration of nausea and vomiting in those randomized to hydroxychloroquine, and one person in the usual care group experienced a serious adverse event.

Median time to recovery in the Usual Care arm during the early phase of the trial when hydroxychloroquine was open to recruitment was shorter (median 9 days) than in the later phases of the trial when the ivermectin and favipiravir arms were open (median 16 days). It is also plausible that those recruited earlier in the trial had less severe illness, which may be partly explained by the lack of easily available testing for SARS-COV-2 in the earlier phase of the pandemic and fewer participants being positive for SARS-COV-2. A secondary analysis of time to recovery based on all participants recruited into the trial up to the point of data lock (30th November 2020) showed an observed difference in median time to recovery of 1 day (7 vs. 8 days) and comparable effect size estimates for the modelled benefit.

We identified four trials of early community treatment not specifically targeting a higher risk population, two of which showed small, non-statistically significant estimates of benefit from hydroxychloroquine. A review of placebo controlled trials found no benefit among outpatients from hydroxychloroquine treatment. A Cochrane review of hydroxychloroquine/chloroquine studies for all COVID-19 primarily in hospitalised patients reported no benefits, but only included trials up to September 2020. The WHO recommends against the use of this drug for COVID-19 infection in all settings.

A more recent systematic review of placebo controlled trials found no benefit among outpatients from hydroxychloroquine treatment^15^. Other recent systematic reviews and an individual patient data meta-analysis found no evidence of effect of hydroxychloroquine on mortality and hospitalisations in non-hospitalised participants^16^.

As with our study, no other trial found a statistically significant difference in the small number of hospitalisations between groups^11,12,18^, and none reported any serious adverse event attributed to taking hydroxychloroquine. The average age of participants in each of the trials was around 40 years, and the proportion of participants with chronic medical conditions was 32%^11^ and 53%^12^. The average age in our trial was 60 years and all participants either had a comorbid condition or were aged 65 or older.

Limitations of our study include the low number of participants that were tested and were confirmed to have SARS-CoV-2 infection. This platform trial commenced in the early stages of the pandemic in the UK and reflects real world care based largely on syndromic management at a time when diagnostic testing was not routinely available in the community. These findings are therefore applicable to health systems where diagnostic testing for COVID-19 are limited or delayed, where treatment decisions have to be made empirically on syndromic presentations, and where participants are not vaccinated. Participants were eligible for up to 14 days after symptom onset if still unwell, and where lab testing was available, this was mainly through self-swabbing and posting the swabs to a central laboratory. Self-swabbing, and longer duration of symptoms in an older population may have resulted in false negative results.

The sample size of our study is less than planned, due to suspension of enrolment into the hydroxychloroquine arm by the regulator after the publication of a study, subsequently withdrawn, that identified harm from hydroxychloroquine treatment of patients hospitalised with COVID-19^19^. Many recruiting clinicians advised the trial team that after this announcement and the findings from the RECOVERY Trial^9^, equipoise no longer existed and they could no longer support randomisation to the hydroxychloroquine arm.

This was an open label trial. Small beneficial effects in recovery parameters may relate to expectations of benefit from this drug in an open-label trial, and that hydroxychloroquine has anti-inflammatory properties which can make people feel better when they have a febrile or an anti-inflammatory condition.

In summary, in this open-label under-powered study we found no evidence that hydroxychloroquine added to usual care reduced admission or death to hospital among people in the community at higher risk of complications with syndromic COVID-19, although numbers were small. Hydroxychloroquine modestly shortened median time to first feeling recovered by two days. There were no serious adverse events identified among those taking hydroxychloroquine.

## Electronic supplementary material

Below is the link to the electronic supplementary material.

Supplementary Material 1

Supplementary Material 2

Supplementary Material 3

## Data Availability

Data are provided within the manuscript and may be available for appropriate uses by correspondence with the corresponding author.

## Declarations

### EudraCT number

2020-001209-22 26/3/2020.

### ISRCTN registry

ISRCTN86534580.

### IRAS number

281958.

### Trial steering committee

Carol Green, Phil Hannaford, Paul Little (Chair), Tim Mustill, Matthew Sydes.

### Data monitoring and safety committee

Deborah Ashby (Chair), Nick Francis, Simon Gates, Gordon Taylor, Patrick White.
